# Reduced body cell mass and functions in lower extremities are associated with mild cognitive impairment and Alzheimer’s dementia

**DOI:** 10.1038/s41598-023-39110-9

**Published:** 2023-08-17

**Authors:** Dieu Ni Thi Doan, Kahye Kim, Boncho Ku, Kun Ho Lee, Jaeuk U. Kim

**Affiliations:** 1 Digital Health Research Division, Korea Institute of Oriental Medicine, Daejeon, South Korea; 2 School of Korean Convergence Medical Science, University of Science and Technology, Daejeon, South Korea; 3 Gwangju Alzheimer’s Disease and Related Dementias (GARD) Cohort Research Center, Chosun University, Gwangju, South Korea; 4 Department of Biomedical Science, Chosun University, Gwangju, South Korea; 5 Dementia Research Group, Korea Brain Research Institute, Daegu, South Korea

**Keywords:** Alzheimer's disease, Nutrition, Risk factors

## Abstract

This study examined the alterations of segmental body composition in individuals with Alzheimer’s pathology (AD), including mild cognitive impairment (MCI) and dementia. A multifrequency bioimpedance analysis (BIA) was used to provide segmental water and impedance variables from 365 cognitively normal (CN), 123 MCI due to AD, and 30 AD dementia participants. We compared the BIA variables between the three groups, examined their correlations with neuropsychological screening test scores, and illustrate their 95% confidence RXc graphs. AD dementia participants were older, more depressive, and had worse cognitive abilities than MCI due to AD and CN participants. Although the BIA variables showed weak partial correlations with the cognitive test scores, we found patterns of an increasing water content in lean mass, increasing extra to intracellular water ratio, and decreasing reactance and phase angle in the lower extremities with effect sizes ranging from 0.26 to 0.51 in the groups of MCI and dementia due to AD compared with CN individuals. The RXc graphs upheld the findings with a significant displacement downward and toward the right, dominantly in the lower extremities. Individuals with AD pathology exhibit a reduced body cell mass or cell strength, an abnormal cellular water distribution, and an overhydration status in lean mass, especially in the lower extremities.

## Introduction

Alzheimer’s disease (AD) is an age-related neurodegenerative disorder that is characterized by a progressive decline in cognition such as memory loss, language difficulty, and behavior changes that are severe enough to interfere with daily life^1^. As the population ages, number of people with dementia due to AD is expected to reach 12.7 million by 2050^2^. Currently, there is no existing effective treatment for AD, meaning that one in three older adults to die with Alzheimer’s or other types of dementia worldwide^2^. Additionally, the long duration of illness before death often requires extensive personal care and can lead to a substantial degradation in quality of life, not only for patients but also for caregivers.

Mild cognitive impairment (MCI) is a transitional phase between normal cognition (CN) and progressive stages of cognitive decline such as dementia. MCI due to AD refers to individuals in the early stage of cognitive impairment with evidence of abnormal beta-amyloid (Aβ) deposition^3^. Due to its continuous but slow progression, it may take several years before MCI develops into AD dementia, with an annual rate of approximately 7 to 8%^4^. Combined therapeutic strategies may help to slow the progression of cognitive decline, especially when applied during the early stages of AD^5,6^. MCI, therefore, has become the critical stage for managing AD and the primary focus of recent research on the AD spectrum.

Due to cognitive deterioration, individuals with dementia and MCI are prone to malnutrition and physical deficits, which provoke alterations in their body composition (BC)^7,8^. Conversely, malnutrition and physical deficits may indirectly cause cognitive impairment via secondary oxidative stress (stress caused by the environment), which triggers inflammation and damages neuronal synapses^9,10^. The relationship between cognition and BC can be further examined using bioimpedance analysis (BIA) method, which measures the obstruction of the electrical alternative current that flows through human tissue/organs represented by resistance (R) and reactance (Xc), and phase angle (PA) to estimate BC^11^. Reductions in Xc and PA suggest a lower body cell mass or cell function, whereas a reduction in R implies a relative increase in body fluids and/or a decrease lean body mass with respect to fat components[^11^, see "Supplementary Materials" for more details]. Among the three, PA has been considered a prognostic marker of malnutrition^12^, frailty^13^, and sarcopenia^14^ in clinical practice. As an inexpensive, noninvasive, and reproducible technique^11^, BIA can be used as a safe and reliable method to investigate BC changes in individuals with MCI or dementia due to AD^15,16^.

The most common findings were that people with AD or severe cognitive decline have a reduced lean body mass, a greater resistance indicating higher fat mass, and a lower reactance and phase angle implying decreased body cell mass^17–20^. Although MCI due to AD is an important phase of the AD continuum, studies of BC changes in this state are still limited. Examining the BC changes in this stage might provide valuable insight into the relationship between BC and early cognitive impairment and therefore allow available therapies to be applied to ease their symptoms.

From the regional BC perspective, previous studies have found a dominant association between cognitive ability and BC changes in the lower extremities. Researchers have reported that reductions in skeletal muscle (strength and mass) and motor function in the lower limbs was associated with a higher risk of cortical Aβ burden in AD and general cognitive decline^21–26^. Our previous publication on MCI individuals upheld these findings in the lower extremities^27^. However, these individuals were not confirmed to have AD pathology and had an increase in lean mass and water volume together with a decrease in body cell strength. Therefore, further studies with segmental BC with a more well-defined AD pathology are needed to justify these observations.

As a follow-up effort to evaluate our findings on the AD continuum, we conducted this study to investigate the changes in segmental BC in individuals with confirmations of Aβ positivity, including the early (MCI due to AD) and late (AD dementia) stages of the disease. We assessed BC changes based on estimated whole-body parameters as well as based on the interpretation from raw bioelectrical parameters of segmental resistance, reactance, and phase angles. Additionally, we employed the bioimpedance vector analysis (BIVA) technique to visualize the relative distribution of the height-normalized resistance and reactance values. The confidence ellipse of bivariate graphs were obtained from the upper and lower extremities for the CN, MCI due to AD, and AD dementia groups after sex stratification.

## Materials and methods

### Participants

In this study, a total of 1134 participants were recruited at the Gwangju Alzheimer’s Disease and Related Dementia (GARD) center (Gwangju City, South Korea) from 2019 to 2021. Each participant or their legal guardian provided written informed consent preceding the study. In accordance with inclusion and exclusion criteria, all individuals had more than three years of education, had no clinical signs of hydration imbalance, and had no medical history or ongoing acute or chronic health conditions such as infections, neurological diseases, mental health instability, and excessive alcohol consumption that interfered with the intended study design. To be a CN individual, baseline magnetic resonance imaging must have not shown an abnormal pattern or atrophy, and a positron emission tomography (PET) scan must have confirmed a negative result of Aβ deposition by visual rating.

MCI people were defined as those who were clinically not demented, had a Clinical Dementia Rating scale of 0.5, and had a Seoul Neuropsychological Screening Battery – second edition (SNSB-II) z score no less than − 1.5 in at least one of the domains. The diagnosis of dementia due to AD was carried out using the criteria of the National Institute of Neurological and Communicative Diseases and Stroke/Alzheimer’s Disease and Related Disorder Association (NINCDS/ADRDA) for probable AD. In contrast, CN individuals were required to have no history or current symptoms of cognitive decline, a Clinical Dementia Rating of zero, and a SNSB-II z score no less than − 1.5 for all tests. All participants were aged between 55 and 90 years and had adequate hearing and vision on neuropsychological examination.

**Figure 1:**
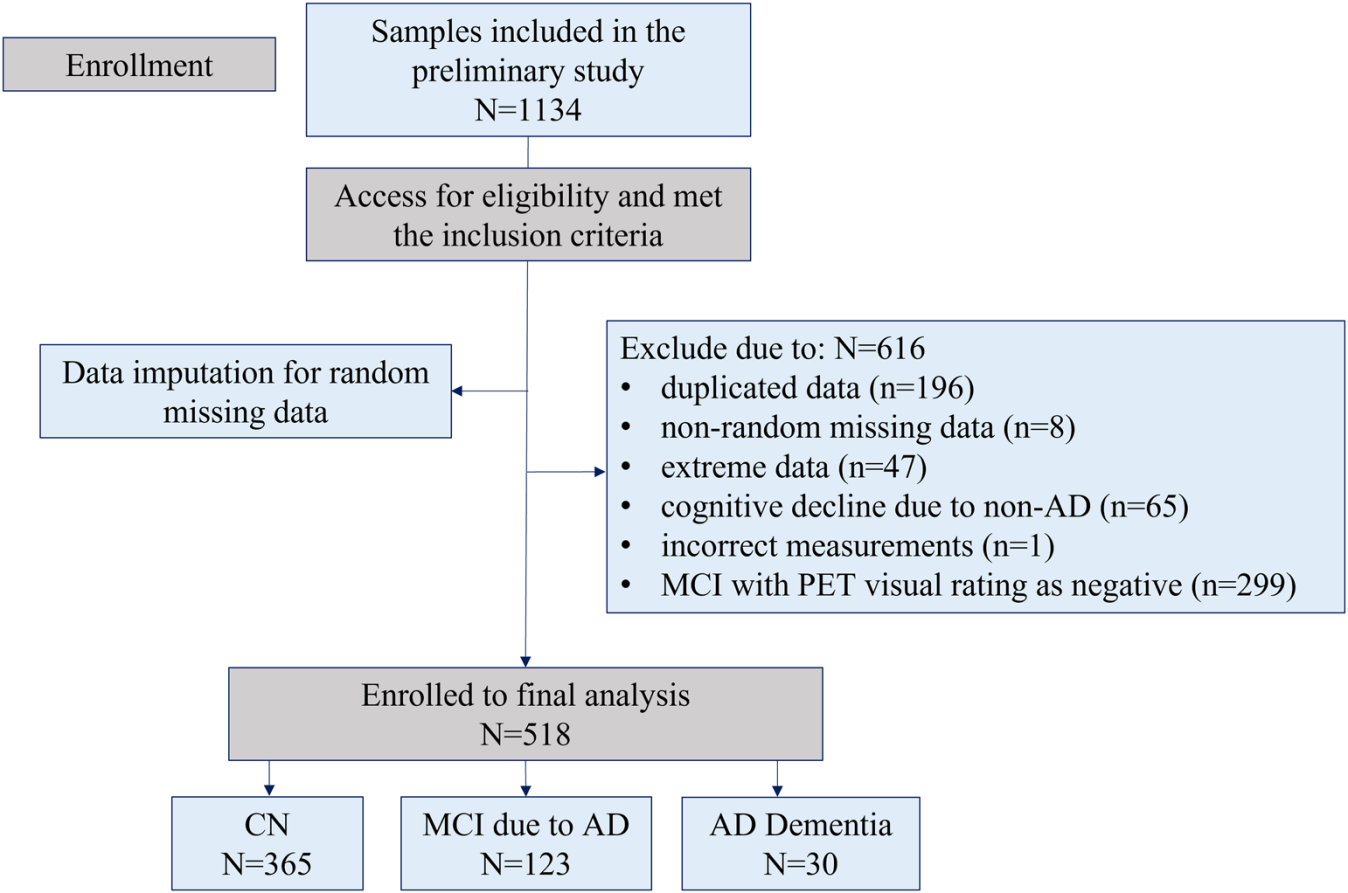
Consolidated Standards of Reporting Trials diagram illustrating enrollment and exclusion criteria for this study.

The included participants then underwent a more precise clinical assessment to obtain demographic information and a medical history alongside a general medical examination, neuropsychological tests, and neurophysiological tests. In the data preprocessing, participants who had nonrandom missing data (n = 8), incorrect BIA measurements (n = 1), extreme results for any variables (n = 47), and/or a diagnosis with any other possible cause of cognitive decline (n = 65) were excluded from the analysis. Furthermore, repeated data obtained from the same participants were also excluded (n = 196). Additionally, MCI individuals who were confirmed to have negative Aβ deposition on the PET scan were excluded from this study (n = 299). A total of 518 participants were included in the final analysis, including 356 CN, 123 MCI due to AD, and 30 AD dementia individuals as illustrated in Fig. 1. The study protocol was approved by the Institutional Review Board of the Chonnam National University Hospital (CNUH; approval number: CNUH-2019–279). The study was performed in agreement with the Declaration of Helsinki.

### Neuropsychological assessment

The Korean version of the Mini-Mental State Examination (K-MMSE) and SNSB-II were used as neuropsychological assessments for all participants. The K-MMSE is a commonly used test for the screening of dementia^28^. It provides a maximum of 30 points, compiling the five cognitive domains of time and spatial orientation, memory, attention and calculation, language, and visuospatial function. The SNSB-II is a comprehensive cognitive test battery that comprises numerous tests to reflect attention, memory, language, visuospatial, and frontal/executive functions and widely used in clinics for evaluating MCI and dementia in South Korea^29,30^. Except for the attention domain, the other four domains of memory, language, visuospatial, and frontal/executive domains were standardized into z-score^31^. Detailed descriptions of these domains can also be found in one of our previous studies^27^. The domain scores of the SNSB-II were adjusted for age, sex, and educational levels^32^. Numerous earlier studies have validated the reliability of the SNSB-II to detect cognitive decline over other cognitive tests, particularly for MCI^30^.

### Anthropometry and bioimpedance measurement

Height (cm), weight (kg), and BMI (kg/m^2^) are the three fundamental anthropometric measurements that were automatically measured from the multifrequency bioimpedance analyzer Inbody S10 Korea^35^. Body composition parameters, including percent body fat mass (PFM = fat mass/weight*100), percent body cell mass (PBCM = body cell mass/weight*100), extracellular water to total body water ratio (ECW/TBW), and basal metabolic rate (BMR), were estimated from impedance variables obtained from the same device. This device uses a tetrapolar 8-point tactile electrode to measure impedance at six electrical frequencies: 1, 5, 50, 250, 500, and 1000 kHz and reactance and phase angle at three frequencies: 5, 50, and 250 kHz. The multifrequency approach was shown to accurately estimate PFM and segmental fat-free mass compared with other methods, such as air displacement plethysmography^36^. The impedance values from each body segment, including the two arms, two legs, and trunk, were then used to estimate segmental intracellular and extracellular water. Participants were examined in a supine position. All measurements were taken by well-trained personnel following the guide of the InbodyS10 manual.

### Bioimpedance vector analysis

An additional BIA approach encompasses vector analysis (BIVA) as developed by Piccoli^37^ was adopted in this study. The relative locations of the ellipses obtained from the three cognitive groups were compared using the height-normalized resistance and reactance variables in the upper and lower extremities with sex stratification. Subsequently, there were four subgroups consisting of men-upper, men-lower, women-upper, and women-lower extremities and three RXc graphs that were created from the CN, MCI due to AD, and AD dementia for each subgroup (Fig. 4 and Table 4).

### Covariates

In previous studies, age and sex were reported as the two factors that could influence body composition^38^ and had relationships with cognitive impairment^39^. Therefore, they were considered two potential covariates to adjust in the multivariate generalized linear model (GLM) analysis in Table 2 and Table 3. Furthermore, as depression is a risk factor of cognitive impairment and AD^40^, we added Geriatric Depression Scale (GDS) as an additional confounder. GDS is a common clinical screening tool to assess depression in older adults that contains thirty questions and provides a maximum score of thirty^41^.

### Statistical analysis

In the data preprocessing and cleaning step, missing values for the SNSB-II domains were imputed using the multiple imputation method. This method was executed using the “mice” function provided by the “mice” package in R software, which helps to complete missing cases by matching with the existing data^42^.

All continuous variables were summarized as the means and standard deviations (SDs); all categorical variables were described as frequencies (n) and proportions (%). For the basic characteristics of participants and neuropsychological test results, a one-way analysis of variance (ANOVA) test was used to compare the means of each continuous variable, and Pearson's chi-squared test or Fisher’s exact test was utilized to examine the independence of each categorical variable between the three groups (CN, MCI due to AD, and AD dementia). For the whole-body composition and segmental variables, we adopted the GLM to investigate the relationship between each of the variables and cognitive groups. Multiple pairwise comparisons between the three cognitive groups were calculated as the estimated marginal mean difference, which was derived from the GLM analysis, and its 95% confidence interval (CI) based on the z-test. Effect sizes of the mean differences were calculated as suggested by Lakens^43^.

In addition to the bivariate correlations between age and neuropsychological test scores with the selected BIA variables, the partial correlation between them upon controlling for age and sex was also investigated using Pearson correlation coefficients.

With the BIVA method, Hotelling’s T^2^ test, a multivariate generalization of the t test, was used to compare the mean impedance vectors of pairwise groups (MCI due to AD vs. CN, AD dementia vs. MCI due to AD, and AD dementia vs. CN), which were graphically visualized using 95% probability confidence ellipses. Mahalanobis distance (MD) was also calculated. Confidence ellipse plots were generated using the classic BIVA software, which was donated by Piccoli and Pastori^44^.

A p value of less than 0.05 was considered statistically significant. R version 4.1.2 (The R Project for Statistical Computing) was used for the statistical analyses.

### Data selection

In this study, in addition to anthropometric measurements such as height, weight, and BMI, four whole-body composition variables were employed, including PBCM, PFM, ECW/TBW, and BMR. As suggested from one of our previous studies, segmental variables in the upper and lower extremities were computed as the average values between the right and left arm and leg^27^. Five pairs of segmental variables were generated, including relative water volume to lean mass (Water_Lean_upper, Water_Lean_lower), extra to intracellular water ratio (ECW_ICW_upper, ECW_ICW_lower), resistance (R_upper, R_lower), reactance (Xc_upper, Xc_lower), and phase angle (PA_upper, PA_lower) in the upper and lower extremities. With the BIVA, height-normalized impedance variables were obtained in the upper and lower extremities by dividing the corresponding resistance or reactance variable by height. A frequency of 50 kHz was used for all impedance results.

## Results

In the results section, MCI was used to denote the MCI due to AD group, and dementia was used to indicate the AD dementia individuals.

### Participants’ characteristics

We described the basic characteristics of the participants in Table 1. Participants in the dementia group was older than the MCI group, whose participants were slightly older than those in the CN group, with mean (SD) ages of 77.0 (7.0), 74.2 (5.4), and 70.9 (5.8), respectively. The educational level was relatively high for all participants, with an average of 12 years in all three groups. In terms of sex, the proportions of males in the dementia, MCI, and CN groups were 66.7%, 56.1%, and 39.5%, respectively. Participants in the dementia group showed worse cognitive functions than the MCI and CN groups as demonstrated by the significantly lower scores in the K-MMSE and the SNSB-II five domains. Additionally, participants in the dementia group showed a higher level of depression than the MCI group, whereas participants in the MCI group were more depressed than the CN group as indicated by GDS scores of 11.8 (8.1), 9.4 (7.1), and 8.0 (6.8), respectively. Finally, all the relevant comorbidities, such as diabetes, hypertension, and hyperlipidemia, were proportionated similarly between the three groups. Notably, all of the participants who encountered these comorbidities were receiving the relevant medications.

**Table 1:** Demographic information and neuropsychological test scores in CN, MCI due to AD, and AD dementia groups.

Variables	Total (n = 518)^1^	CN (n = 365)^1^	MCI due to AD (n = 123)^1^	AD dementia (n = 30)^1^	*p* value^2^
Age [year]	72.1 (6.1)	70.9 (5.8)	74.2 (5.4)	77.0 (7.0)	**< 0.001**
Male, n (%)	233 (45.0%)	144 (39.5%)	69 (56.1%)	20 (66.7%)	**0.001**
Education level [year]	12.2 (4.5)	12.2 (4.5)	12.3 (4.6)	11.6 (4.4)	0.725
Systolic BP [mmHg]	126.7 (14.4)	127.3 (14.8)	124.8 (13.2)	126.8 (14.9)	0.253
Diastolic BP [mmHg]	71.8 (9.9)	72.1 (10.3)	71.1 (8.7)	70.9 (9.3)	0.525
GDS	8.6 (7.0)	8.0 (6.8)	9.4 (7.1)	11.8 (8.1)	**0.005**
K-MMSE	26.8 (2.9)	27.6 (2.0)	26.0 (2.7)	20.8 (4.9)	**< 0.001**
*SNSB II*					
Attention	9.3 (2.3)	9.7 (2.3)	8.4 (2.0)	8.1 (1.9)	**< 0.001**
Language	0.1 (0.4)	0.2 (0.3)	− 0.1 (0.5)	− 0.6 (1.0)	**< 0.001**
Visuospatial	0.4 (0.6)	0.6 (0.3)	0.2 (0.7)	− 0.6 (1.2)	**< 0.001**
Memory	0.0 (0.8)	0.3 (0.5)	− 0.6 (0.7)	− 1.7 (0.5)	**< 0.001**
Frontal	0.1 (0.7)	0.3 (0.5)	− 0.4 (0.7)	− 1.3 (0.6)	**< 0.001**
Comorbidities					
Diabetes	95 (18.3%)	62 (17.0%)	25 (20.3%)	8 (26.7%)	0.340
Hypertension	225 (43.4%)	153 (41.9%)	57 (46.3%)	15 (50.0%)	0.524
Hyperlipidemia	100 (19.3%)	79 (21.6%)	19 (15.4%)	2 (6.7%)	0.063
Thyroid	19 (3.7%)	11 (3.0%)	7 (5.7%)	1 (3.3%)	0.329
Circulatory disorder	80 (15.5%)	51 (14.0%)	26 (21.1%)	3 (10.0%)	0.133
Osteoarthritis	160 (30.9%)	119 (32.6%)	36 (29.5%)	5 (16.7%)	0.178

*BP* blood pressure; *GDS* geriatric depression scale; *K-MMSE* Korean version of mini-mental state examination; *SNSB-II* second edition of seoul neuropsychological screening battery.

^1^The values represent mean (SD) for continuous variables and n (%) for categorical variables.

^2^The *p* values for the continuous variables were obtained from one-way analysis of variance (ANOVA) test. For the categorical variable, the *p* values were derived from the Pearson's Chi-squared test or Fisher’s exact test.

Bold text indicates a *p* value < 0.05.

### Anthropometric and Whole-Body Composition

Table 2 describes the anthropometric measurements such as height, weight, and BMI and the whole-body composition results in the three cognitive groups. Among these variables, only the ECW/TBW was significantly higher in the MCI group than in the CN group. The mean difference was small ($\overline{\Delta }$ = 0.177, *p* < 0.05), and the effect size was relatively small, with a $\Gamma$ (gamma) of 0.29. Overall, our participants in the CN, MCI, and dementia groups had similar body sizes and whole-body compositions as indicated by the insignificant differences between groups in variables such as height, weight, BMI, PFM, and PBCM.

**Table 2:** Anthropometry and whole-body composition in CN, MCI due to AD, and AD dementia groups upon controlling for age, sex, and GDS score.

Variables	Total (n = 518)^1^	CN (n = 365)^1^	MCI due to AD (n = 123)^1^	AD dementia (n = 30)^1^	MCI due to AD versus CN		AD dementia versus MCI due to AD		AD dementia versus CN	
					$\overline{\Delta }$(95% CI)	$\Gamma$	$\overline{\Delta }$(95% CI)	$\Gamma$	$\overline{\Delta }$(95% CI)	$\Gamma$
Height [cm]	159.0 (8.5)	158.5 (8.2)	160.3 (9.0)	159.4 (8.6)	0.435 (− 0.867, 1.736)	0.09	− 1.353 (− 3.822, 1.116)	0.26	− 0.918 (− 3.297, 1.460)	0.18
Weight [kg]	62.8 (9.6)	62.8 (9.4)	63.0 (10.5)	61.6 (6.9)	− 0.475 (− 2.504, 1.554)	0.06	− 1.527 (− 5.376, 2.322)	0.19	− 2.002 (− 5.710, 1.706)	0.25
BMI [kg/m^2^]	24.8 (2.9)	24.9 (2.8)	24.5 (3.3)	24.2 (1.9)	− 0.328 (− 1.058, 0.403)	0.11	− 0.149 (− 1.535, 1.237)	0.05	− 0.477 (− 1.812, 0.858)	0.17
Body cell mass [%]	42.9 (5.0)	42.6 (5.0)	43.7 (5.0)	43.1 (5.3)	0.421 (− 0.536, 1.377)	0.11	− 0.993 (− 2.808, 0.822)	0.26	− 0.572 (− 2.321, 1.177)	0.15
Fat mass [%]	33.2 (7.6)	33.8 (7.5)	31.8 (7.6)	32.7 (7.9)	− 0.825 (− 2.305, 0.655)	0.14	1.590 (− 1.218, 4.398)	0.27	0.765 (− 1.940, 3.470)	0.13
ECW/TBW	39.3 (0.7)	39.2 (0.7)	39.5 (0.8)	39.7 (0.6)	0.177* (0.024, 0.330)	0.29	0.053 (− 0.237, 0.342)	0.09	0.229 (− 0.050, 0.509)	0.38
Basal metabolic rate [kcal]	1,275 (173)	1,268 (171)	1,297 (179)	1,268 (160)	3.193 (− 20.98, 27.36)	0.03	− 38.449 (− 84.31, 7.41)	0.40	− 35.257 (− 79.43, 8.92)	0.37

*ECW/TBW* extracellular to total body water ratio.

Mean (SD) of the continuous variables were presented. The mean difference ($\overline{\Delta })$ (and 95% confidence intervals) between groups were analyzed using estimated marginal means comparison from Generalized Linear Model upon controlling for age, sex, and GDS score. *P* values were adjusted using multiplicity adjustment Tukey test and denoted by * *p* < 0.05, ** *p* < 0.01, *** *p* < 0.001. Effect sizes (Γ) were calculated as suggested by Lakens (2013)^41^. Bold text indicates a significant mean difference with *p* < 0.05.

### Descriptions of the segmental body composition and bioimpedance variables

The descriptions of segmental bioimpedance variables and their comparisons between CN, MCI, and dementia are shown in Table 3. We found significant differences between the CN and MCI groups in the Water_Lean_lower, ECW_ICW_lower, Xc_lower, and PA_lower variables. The effect sizes obtained from these variables were mild and ranged from 0.26 to 0.37. Notably, the results of statistical p values did not correspond to the effect sizes due to the difference in the sample sizes^45^, as we saw here between dementia and either MCI or CN. Between dementia and CN groups, the effect sizes obtained from these variables were ranged from 0.34 to 0.51.

### Visualization by boxplot

**Figure 2:**
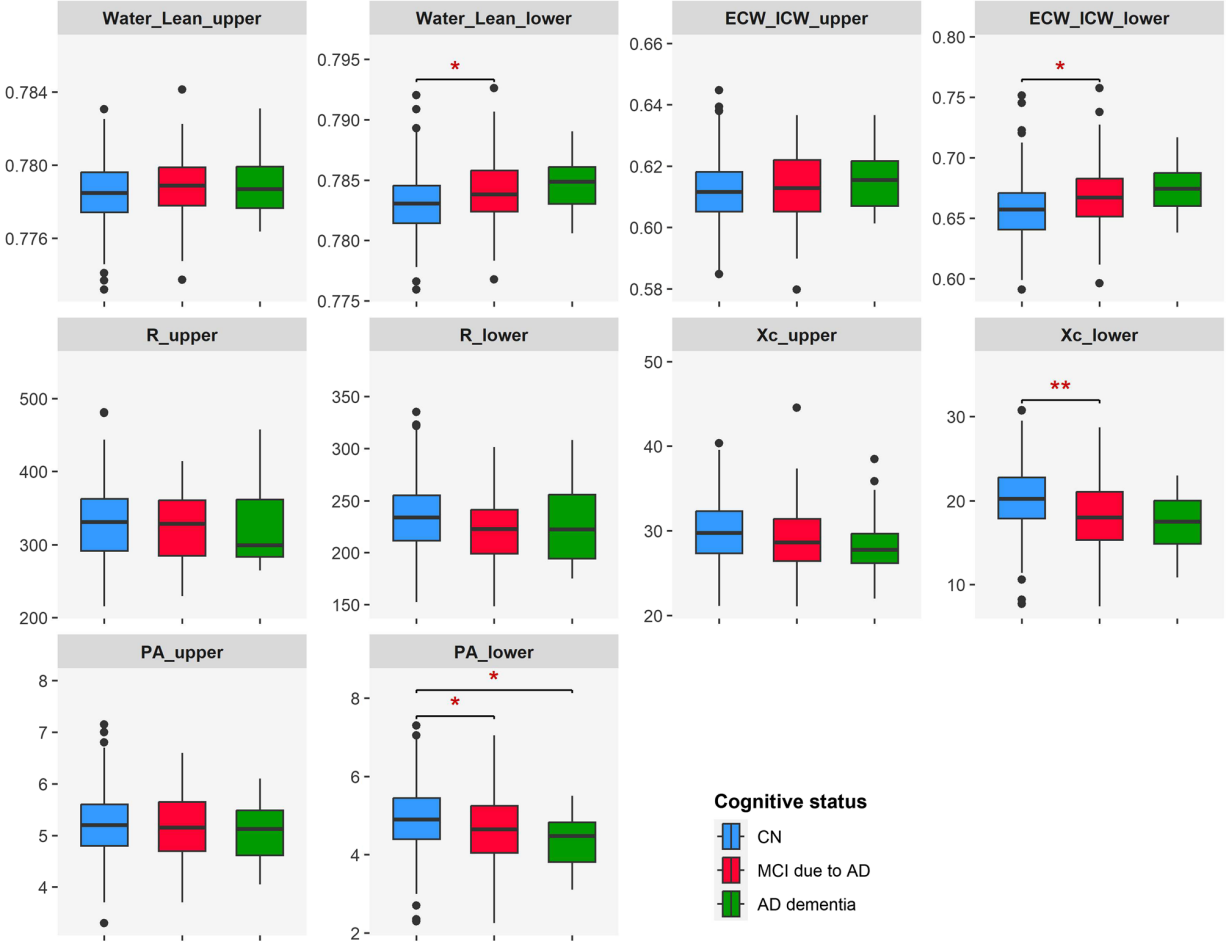
Boxplot to visualize the distributions of segmental bioimpedance variables in CN, MCI due to AD, and AD dementia groups upon controlling for age, sex, and GDS score. *p < 0.05, **p < 0.01, ***p < 0.001. Comparisons with non-significant results are not displayed.

To illustrate the alterations in segmental bioimpedance variables between the three groups, CN, MCI, and dementia, we provided Fig. 2. As described in Table 3, we found a trend of increasing Water_Lean_lower and ECW_ICW_lower and decreasing Xc_lower and PA_lower from the CN to MCI and to dementia groups. Nonetheless, these patterns were more clearly observed in the variables related to the lower extremities.

**Table 3:** Segmental bioimpedance variables in CN, MCI due to AD, and AD dementia groups upon controlling for age, sex, and GDS score.

Variables	Total (n = 518)^1^	CN (n = 365)^1^	MCI due to AD (n = 123)^1^	AD dementia (n = 30)^1^	MCI due to AD versus CN		AD dementia versus MCI due to AD		AD dementia versus CN	
					$\overline{\Delta }$(95% CI)	$\Gamma$	$\overline{\Delta }$(95% CI)	$\Gamma$	$\overline{\Delta }$(95% CI)	$\Gamma$
Water_Lean_upper	0.779 (0.002)	0.779 (0.002)	0.779 (0.002)	0.779 (0.002)	0.000 (− 0.000, 0.001)	0.09	− 0.000 (− 0.001, 0.001)	0.08	0.000 (− 0.001, 0.001)	0.00
Water_Lean_lower	0.783 (0.002)	0.783 (0.002)	0.784 (0.003)	0.785 (0.002)	0.001 * (0.000, 0.001)	**0.29**	0.000 (− 0.001, 0.001)	0.15	0.001 (− 0.000, 0.002)	**0.44**
ECW_ICW_upper	0.613 (0.010)	0.612 (0.010)	0.614 (0.011)	0.616 (0.009)	0.000 (− 0.002, 0.003)	0.04	0.001 (− 0.004, 0.005)	0.09	0.001 (− 0.003, 0.006)	0.13
ECW_ICW_lower	0.661 (0.024)	0.657 (0.023)	0.668 (0.026)	0.675 (0.021)	0.006 * (0.001, 0.011)	**0.30**	0.002 (− 0.008, 0.012)	0.10	0.008 (− 0.001, 0.018)	**0.41**
R_upper	328 (48)	330 (48)	324 (46)	322 (52)	3.148 (− 5.337, 11.632)	0.09	2.498 (− 13.598, 18.594)	0.07	5.645 (− 9.861, 21.152)	0.17
R_lower	232 (33)	235 (33)	222 (33)	228 (36)	− 7.377 (− 14.789, 0.036)	0.25	10.024 (− 4.039, 24.087)	0.34	2.648 (− 10.901, 16.196)	0.09
Xc_upper	29.6 (3.7)	29.9 (3.7)	29.1 (3.9)	28.3 (3.7)	− 0.035 (− 0.900, 0.831)	0.01	− 0.223 (− 1.865, 1.419)	0.07	− 0.258 (− 1.840, 1.324)	0.08
Xc_lower	19.6 (4.0)	20.3 (3.8)	18.0 (4.0)	17.2 (3.3)	− 1.245 ** (− 2.111, − 0.379)	**0.37**	0.084 (− 1.559, 1.727)	0.02	− 1.161 (− 2.744, 0.421)	**0.34**
PA_upper	5.21 (0.61)	5.23 (0.61)	5.17 (0.62)	5.08 (0.58)	− 0.058 (− 0.180, 0.065)	0.12	− 0.070 (− 0.303, 0.163)	0.15	− 0.128 (− 0.352, 0.097)	0.27
PA_lower	4.85 (0.82)	4.95 (0.81)	4.66 (0.83)	4.36 (0.68)	− 0.169 * (− 0.337, − 0.001)	**0.26**	− 0.171 (− 0.489, 0.148)	0.26	− 0.340* (− 0.647, − 0.033)	**0.51**

The methodological details are identical to those presented in the footer of Table 2. Bold text indicates a significant mean difference with *p* value < 0.05 and dominant effect sizes (Γ).

*ECW_ICW* extracellular to intracellular water ratio; *R* resistance; *Xc* reactance; *PA* phase angle.

### Correlations between age, neuropsychological test scores, whole-body composition and segmental bioimpedance variables

**Figure 3:**
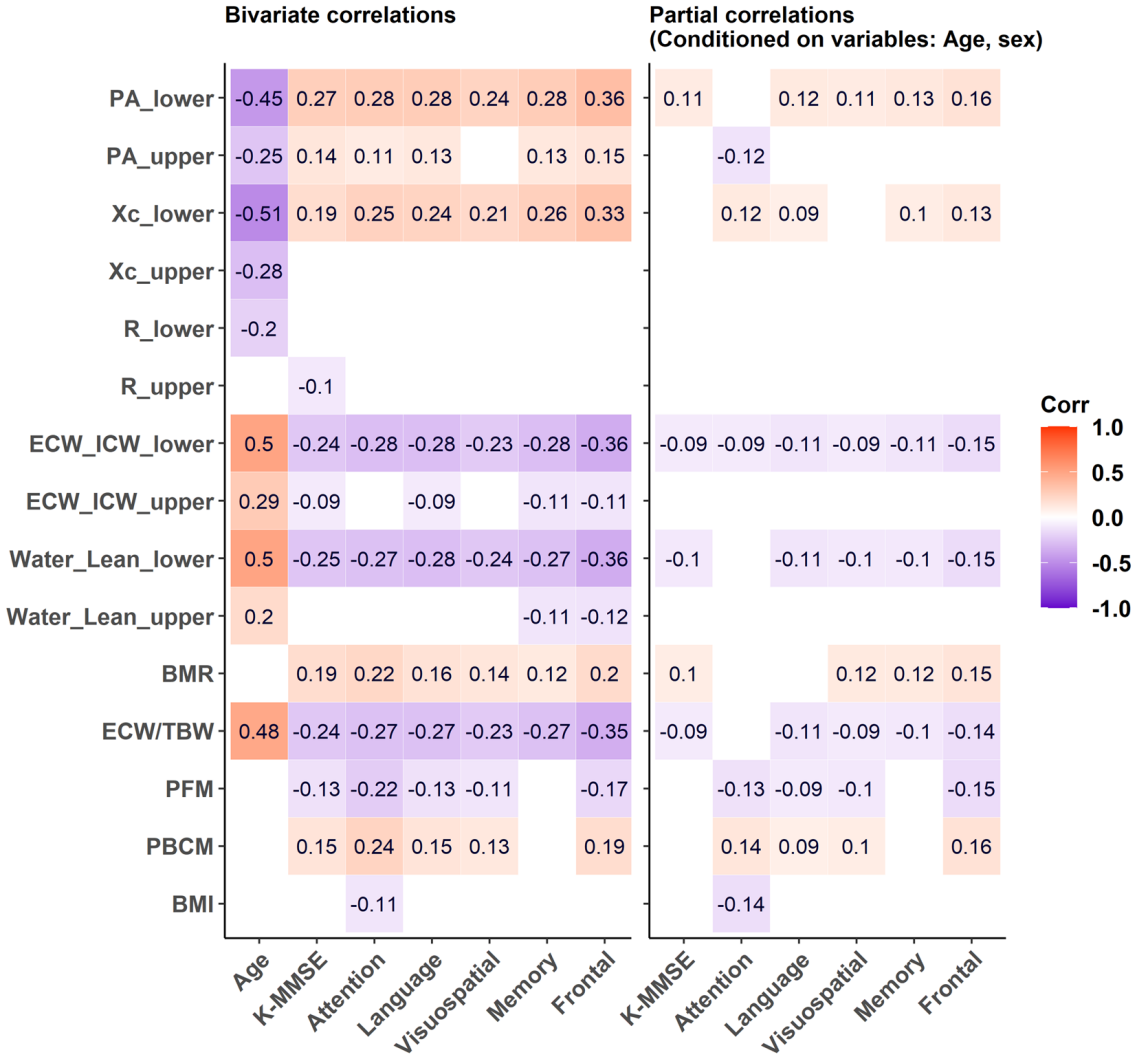
Correlations between the selected BIA variables and neuropsychological tests before and after controlling for age and sex. Empty cells: insignificant correlation coefficients (*p* value > 0.05).

In Fig. 3, the correlations between the selected BIA variables and age and neuropsychological tests are illustrated.

Notably, age showed positive correlations with ECW/TBW, Water_Lean_lower, and ECW_ICW_lower and negative correlations with the Xc_lower and PA_lower variables with correlation coefficients r ranging from 0.45 to 0.51 (absolute value). These correlations were weaker for the upper extremity variables.

The BIA variables showed weak to moderate correlations with the cognitive test scores. After taking age and sex into account, most of the correlations diminished. Frontal-executive function showed the best partial correlation with those variables in the lower extremities, with the highest absolute coefficient of 0.16.

### Segmental bioimpedance vector analysis

**Figure 4:**
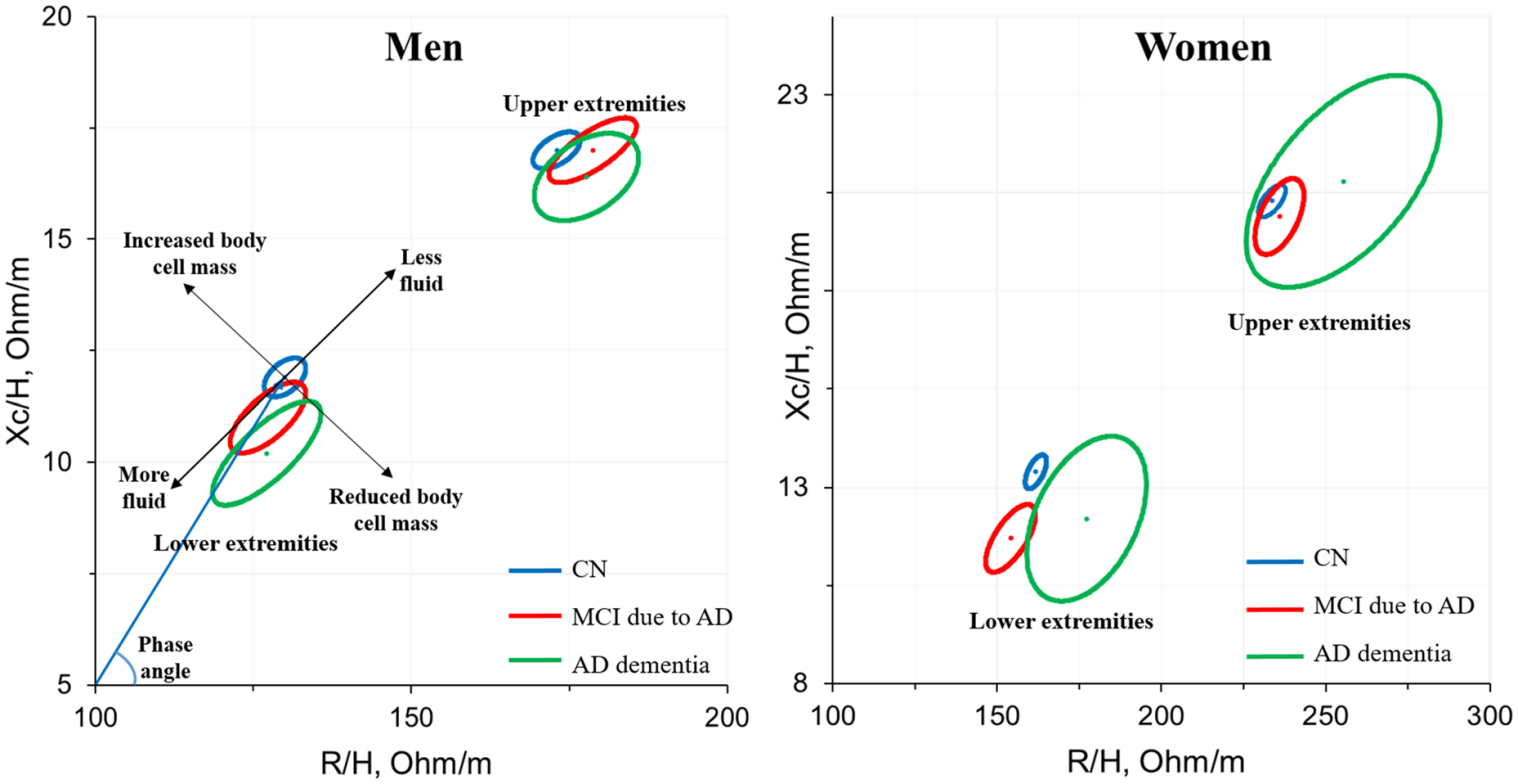
Relative locations of 95% confidence segmental RXc ellipses in the upper and lower extremities for women and men with CN, MCI due to AD, and AD dementia.

The relative locations of the 95% confidence ellipses representing RXc graphs obtained from the CN, MCI, and dementia individuals are illustrated in Fig. 4. As comparisons were made using variables in the upper and lower extremities for men and women separately, there were four subgroups containing three ellipses for each group, as mentioned above. We found that in all four subgroups, the RXc graphs demonstrated right-side migration patterns, indicating a lower body soft mass in the dementia group than in the CN group, and the MCI graphs were located between them. The details of the shifted distances are described in Table 4. In comparison to the upper extremities, the RXc graphs in the lower extremities exhibited more obvious displacements toward a lower body soft mass with the Mahalanobis Distance of 0.86 and 1.42 between dementia versus CN in men and women, respectively.

**Table 4:** Evaluation of the distance between every two ellipses obtained from the three cognitive groups for men and women.

	Variables	MCI due to AD versus CN		AD dementia versus MCI due to AD		AD dementia versus CN	
		MD	T^2^	MD	T^2^	MD	T^2^
Men	R/H_upper	0.37*	6.4	0.33	1.7	0.59*	6.2
	Xc/H_upper						
	R/H_lower	0.40*	7.6	0.44	3	0.86**	12.9
	Xc/H_lower						
Women	R/H_upper	0.30	3.8	0.86	6.3	0.98*	9.1
	Xc/H_upper						
	R/H_lower	0.66***	18.9	1.27**	13.6	1.42***	19.4
	Xc/H_lower						

Mahalanobis Distance (MD) demonstrated the distance between the bivariate data of two cognitive groups (assuming normal distribution) with the evaluation of statistical significance using Hotelling’s T^2^ test. Presented in boldface for the MD with *p < 0.05, **p < 0.01, ***p < 0.001.

R/H and Xc/H represent height-normalized resistance and reactance.

## Discussions

The results of this study revealed that while whole-body composition did not differ between the CN, MCI due to AD, and AD dementia groups, there were significant changes in segmental BC in individuals with confirmed AD pathology. Specifically, the water content in lean mass and the cellular water ratio in the lower extremities tended to increase with worsening cognitive impairment, while cellular health indicators such as Xc and PA tended to decrease. These findings suggest that a lower body cell mass or strength and abnormal water distribution may be related to cognitive impairment due to AD. With the BIVA method, the shifting of the impedance vectors of the MCI and dementia due to AD groups toward the right side of the CN ellipses strengthened the lower soft mass observation. The overhydration status was observed mainly in the lower extremities in women or men with MCI due to AD and in men with AD dementia.

To the best of our knowledge, this is the first study to describe the segmental BC changes in individuals with an AD continuum. The association between the reduction in whole-body soft tissue and cognitive decline, such as MCI and AD has been reported^17,18,20,46^ but not segmentally. Martin and colleagues found that the RXc graphs of the mixed-type dementia group significantly shifted toward the right side of the RXc graphs in the nondementia individuals^19^. Cova and the team also reported similar displacement in the dementia and MCI groups^20^. Buffa et al. emphasized a more accentuated lean mass reduction in women with worse cognitive status^17,46^. Our findings are in agreement with these studies.

Previous studies have reported an increase in the ECW_ICW ratio in the dementia group compared with the cognitively normal group^19^. This increase can be explained by the correlations between ECW_ICW and age and cognitive impairment pathology^47,48^. During aging, cell deterioration may cause abnormal cellular permeability, thus increasing ECW_ICW. Moreover, ECW_ICW and cognitive impairment may share common pathological pathways, such as systemic inflammation, hypertension, and blood‒brain barrier permeability^48^. Upon controlling for age, sex, and GDS score, we observed an increase in ECW_ICW across the cognitive groups on the AD continuum, especially in the lower extremities. This suggested a better sensitivity for detecting small changes in cellular water distribution in the AD pathology from the lower extremities compared with the upper extremities.

The water lean ratio is another useful indicator to assess the lean mass hydration status, which may serve the same interpretation as total the body water per fat-free mass ratios. An excessive volume of extracellular fluid induces edema, which is typically caused by chronic diseases such as heart failure and renal dysfunction^49,50^. These medical conditions may also be risk factors for cognitive decline due to the reduction in cerebral blood flow, the change in blood brain barrier^51^, the lower glomerular filtration rate, or the presence of albuminuria^52^. An increasing Water_Lean_lower along with the worsening cognitive stages suggested a lean mass overhydration status in the lower extremities in relation to AD pathology.

In a negative correlation with ECW_ICW, phase angle (PA) has been considered a prognostic marker of several clinical conditions^12–14,53^. Similar to ECW_ICW, PA is also an indicator of inflammation and cell membrane integrity^54^. A decrease in PA was also found in dementia and mild-moderate AD^19,46^. Furthermore, Cova and colleagues monitored BC changes based on vectorial parameters such as PA in individuals with mild to moderate AD for approximately nine months of follow-up^55^. They found that although PA and other BC variables appeared to be helpful markers in prodromal phases such as MCI due to AD, they remained stable or slowly altered in the later stage of the disease. Our findings are in agreement with this study as we found that the magnitude of differences or effect sizes between the AD dementia and MCI due to AD groups was not as great as those between the MCI due to AD and CN groups. In other words, the reduction in body cell mass or cell strength was exclusively manifested in the early stage of the disease rather than in the later stages. The early systemic manifestation of the pathological process may be the cause for this observation. Nevertheless, we still observed similar patterns across the attenuations of cognitive impairment to the later stages, albeit at a slower pace.

Reactance is another parameter that reflects the ability of the cell membrane to hold charges^17^. Reductions in reactance or height-normalized reactance have been reported in dementia or women with MCI or with worse psychofunctional status^18,19,27^. Lower reactance indicates lower cell membrane health or cell strength and thus weaker obstruction of the charges in individuals with dementia and MCI due to AD.

Notably, resistance variables in the upper and lower extremities did not form consistent patterns along with the degree of cognitive deterioration. Our previous study suggested a significant reduction in R_lower in the MCI stage, indicating a lower fat mass with respect to fat-free mass^27^. However, when examining the later stage of the AD continuum, this observation was no upheld. Lower fat mass has been found to positively correlate with lower cognitive function in the elderly Korean population^56,57^, and a decrease in fat mass is associated with a higher risk of MCI, particularly in women with ApoE-e4^58^. Similarly, unintended weight loss or BMI reduction might be the preceding markers of cognitive impairment^33^. On the other hand, overweight or obese populations have a lower risk of developing AD^33^. These studies suggested that fat mass measurements decrease in the early stage and increase in the later stage of cognitive impairment. Therefore, we assume that resistance variables have a U-shaped relationship with the progression of cognitive decline due to AD.

The BIVA method takes into account the covariance relationship between height-normalized resistance and reactance, allowing for different interpretations of BC for each subgroups based on the transforming migration directions of these RXc graphs. Overall, all subgroups showed significantly lower body soft tissue in the AD dementia or MCI due to AD groups compared to the CN groups, which supports our findings using segmental BIA variables. In a previous study, Burns and colleagues found negative associations between whole-brain volume, white matter volume, and global cognitive performance with lean body mass, regardless of age and sex^34^. Similarly, higher lean mass, particularly muscle mass in the thighs, was associated with a lower risk of Aβ positivity in women^34^. Therefore, reduced lean mass might precede cognitive decline due to AD. With regard to hydration, men with MCI and dementia due to AD exhibited a more fluid condition in the lower extremities, and women with MCI due to AD showed a similar result, but women with AD dementia appeared to have a less fluid status compared to the CN in their subgroups. Buffa et al. previously reported a dehydration status in women with severe AD with respect to mild-moderate AD^17^. The hydration status of our participants varied across cognitive stages and gender. Further longitudinal studies are needed to monitor this condition.

Lower extremities seemed to play an important role in regional BC changes related to AD pathology. We presume that due to the relatively greater volume of body mass in the lower extremities compared with the upper extremities, its BC changes in the lower extremities were more sensitively recognized. Furthermore, lower extremities might be influenced by immobility or physical activity attenuations more than upper extremities. Therefore, more focus on the lower extremities might be needed when examining BC changes in individuals with the AD continuum.

In this study, we were able to examine the segmental BC changes in individuals with AD pathology, including the early stage of MCI and the later stage of dementia. With refined inclusion criteria, the results were established based on the AD continuum, which has not been reported before to the best of our knowledge. Once again, we observed that the segmental variables in the lower extremities play a more crucial role in the relationship with cognitive impairment due to AD than those in the upper extremities. These BC changes may share similar pathological pathways with the cognitive impairment of AD. Thus, these variables might be considered potential markers in the screening of cognitive decline due to AD.

There were some limitations in this study that need to be addressed. First, the number of demented participants was dramatically smaller than those of the MCI due to AD and CN groups. This imbalance in sample size might influence the comparisons in the pairwise analysis, and a larger amount of AD dementia data might generate statistically significant results when compared with the MCI due to AD and CN groups. Second, although food intake and physical exercise affect BC, we did not have access to the nutritional status or daily activity hours of the participants. Finally, longitudinal studies with substantial and balanced sample sizes are needed to validate our findings on the progression of cognitive decline due to AD.

## Conclusion

Although the selected segmental BIA variables did not show a strong correlation with the neuropsychological test scores upon controlling for age and sex, they were demonstrated as useful indicators in examining the regional BC changes in individuals with AD pathology, early stage as MCI, and later stage as dementia. An increase in water content in lean mass, an abnormal cellular water distribution, and a reduction in body cell mass or cell strength are significantly related to the cognitive impairment stages due to AD, especially in the lower extremities. These characteristics of segmental BIA variables may serve as additional potential markers in the diagnosis of the AD continuum. Further studies are needed to validate the hydration status in different stages of cognitive decline due to AD.

### Supplementary Information

Supplementary Information.

## Data Availability

The original contributions presented in the study are included in the article, further inquiries can be directed to the corresponding author.
