# Supplementary material for: Reduced body cell mass and functions in lower extremities are associated with mild cognitive impairment and Alzheimer’s dementia
Source: Sci Rep. 2023 Aug 17;13:13389. doi: 10.1038/s41598-023-39110-9 (PMC10435546; doi:10.1038/s41598-023-39110-9)
Supplement: Supplementary file 1 — Supplementary Information. [file 41598_2023_39110_MOESM1_ESM.docx]

**Reduced Body Cell Mass and Functions in Lower Extremities are Associated with Mild Cognitive Impairment and Alzheimer’s Dementia**

**Dieu Ni Thi Doan^1,2^, Kahye Kim^1^, Boncho Ku^1^, Kun Ho Lee^3,4,5^, and Jaeuk U. Kim^1,2,*^**

^1^Digital Health Research Division, Korea Institute of Oriental Medicine, Daejeon, South Korea

^2^School of Korean Convergence Medical Science, University of Science and Technology, Daejeon, South Korea

^3^Gwangju Alzheimer’s Disease and Related Dementias (GARD) Cohort Research Center, Chosun University, Gwangju, South Korea

^4^Department of Biomedical Science, Chosun University, Gwangju, South Korea

^5^Dementia Research Group, Korea Brain Research Institute, Daegu, South Korea

***Correspondence:** Jaeuk U. Kim, jaeukkim@kiom.re.kr

Supplementary Materials

**Supplementary Table S1.** Description of the whole-body and segmental variables considered in this study.

| **Variable** | **Description** | **Unit** | **Definition** | |
| --- | --- | --- | --- | --- |
| PFM | Percent fat mass | % | Fat mass/Weight *100 | |
| PBCM | Percent body cell mass | % | Body cell mass/Weight*100 | |
| ECW/TBW | Extracellular to total body water ratio | _ | ECW/TBW | |
| BMR | Basal metabolic rate | Kcal |  | |
| Water_Lean_upper | Ratio of average segmental water to average segmental lean on upper limbs | _ | $\frac{SW\_RA+SW\_LA}{SL\_RA+SL\_LA}$ | |
| Water_Lean_lower | Ratio of average segmental water to average segmental lean on lower limbs | _ | $\frac{SW\_RL+SW\_LL}{SL\_RL+SL\_LL}$ | |
| ECW_ICW_upper | Average of segmental extra-intracellular water ratio on upper limbs | _ | ($\frac{ECW\_TBW\_RA}{1-ECW\_TBW\_RA}$ + $\frac{ECW\_TBW\_LA}{1-ECW\_TBW\_LA}$)/2 | |
| ECW_ICW_lower | Average of segmental extra-intracellular water ratio on lower limbs | _ | ($\frac{ECW\_TBW\_RL}{1-ECW\_TBW\_RL}$ + $\frac{ECW\_TBW\_LL}{1-ECW\_TBW\_LL}$)/2 | |
| R_upper | Average of segmental resistance on upper limbs at 50 kHz | Ohm | $\frac{\sqrt{{Z\_RA}^{2}-{Xc\_RA}^{2}}+\sqrt{{Z\_LA}^{2}-{Xc\_LA}^{2}}}{2}$ | |
| R_lower | Average of segmental resistance on lower limbs at 50 kHz | Ohm | $\frac{\sqrt{{Z\_RL}^{2}-{Xc\_RL}^{2}}+\sqrt{{Z\_LL}^{2}-{Xc\_LL}^{2}}}{2}$ | |
| Xc_upper | Average of segmental reactance on upper limbs at 50 kHz | Ohm | $\frac{Xc\_RA+Xc\_LA}{2}$ | |
| Xc_lower | Average of segmental reactance on lower limbs at 50 kHz | Ohm | $\frac{Xc\_RL+Xc\_LL}{2}$ | |
|  | Phase angle at 50 kHz | Degree | tan^-1^ ($\frac{\mathrm{Xc}}{sqrt(Z^2-Xc^2)})*180/\pi$ | |
| PA_upper | Average of segmental phase angle on upper limbs at 50 kHz | Degree | $\frac{PA\_RA+PA\_LA}{2}$ | |
| PA_lower | Average of segmental phase angle on lower limbs at 50 kHz | Degree | $\frac{PA\_RL+PA\_LL}{2}$ | |
| RA: Right arm, LA: Left arm, RL: Right leg, LL: Left leg, SW: Segmental water, SL: Segmental lean, ICW: Intracellular water, ECW: Extracellular water, TBW: Total body water, BMR: Basal metabolic rate, Z: Impedance, Xc: Reactance, R: Resistance, PA: Phase angle – These parameters were directly derived from InbosyS10. | | | |  |

**Supplementary information on BIA electrical parameters and RXc graph in BIVA method**

BIA is a measure of the obstruction to the electrical alternative current that flows through the human tissue/organs represented by impedance (Z) which is composed of resistance and reactance [Kasap 1997]. This technique works based on the assumption that the human body is a homogenous conductive cylinder and its impedance quotient has an empirical relationship with the volume of electrolyte water contains in fat-free mass [Khalil et al., 2014, Kyle et al., 2004]. Resistance is a measure of resistivity and is inversely proportional to the relative amount of total body water. Reactance is the capacitance initiated from cell membranes, indicating the cell mass, volume or strength. Phase angle is another parameter that can be calculated based on resistance and reactance representing the phase shift between voltage and current indicating the cell membrane ability in holding charges [Khalil et al., 2014]. A reduction in reactance and phase angle suggests a lower body cell mass or cell function, whereas a reduction in resistance implies a relative increase in body fluids and/or lean body mass with respect to fat components [Kyle et al. 2004, Tanaka et al., 2019]. Among these three parameters, reduced PA has been considered a prognostic marker of malnutrition [Kubo et al., 2021], frailty [Tanaka et al., 2019], and sarcopenia [Hirose et al., 2020] in clinical practice.

The analysis of bioimpedance that is examined at more than two frequencies is known as multi-frequency bioimpedance analysis. Multi-frequency BIA encompasses impedances at different frequencies (i.e. 5 kHz, 50 kHz, 250 kHz, or 500 kHz) in the linear regression models for the evaluation of fat mass, lean mass, or fluid levels. Since low frequency (i.e. 1 kHz) current cannot penetrate the cell membrane while high frequency (i.e. 500kHz) can pass through the cellular environment, multi-frequency BIA approach can evaluate relative compositions more precisely than single-frequency BIA by considering gender, age, height and weight, especially for extracellular fluid [Khalil et al., 2014, Raimann et al., 2014]. Together with multi-frequency BIA, segmental BIA considers body segments, including arms, legs, and trunk, as five separate cylinders and evaluates each segmental measurement independently [Inbody S10 User’s Manual]. The resistance of a particular body segment differs depending on its cross-sectional area and length, and this value also changes according to the material properties of each segment, such as local water retention, dehydration status, or fat (fat-free) mass distributions [Kyle et al., 2004]. Multi-frequency segmental BIA is superior to single-frequency whole-body BIA in segmentally estimating body composition changes in a clinical setting [Olde Rikkert et al., 1997].

Bioelectrical impedance vector analysis (BIVA) is a variant of the BIA method that uses normalized bioelectrical variables (resistance and reactance) to plot a RXc graph directly for the purpose of comparing between populations [Piccoli et al., 1994]. Classic BIA corrects resistance and reactance for an individual's height to remove the conductor's length. Specific BIA normalizes not only for height but also for transverse areas to minimize the influence of body dimensions [Rossini-Venturini et al., 2022]. The shifting of the RXc vector to the right side indicates a reduction and to the left side implies an increment of body cell mass or cell membrane health. The shifting of the RXc vector downward demonstrates an increase in body fluid, and upward suggests a reduction of body fluid. The illustration of a classic BIVA RXc graph is presented below. Although limited, this method has been utilized in recent research to examine changes in body composition and bioelectrical variables in AD dementia or MCI subjects [Buffa et al., 2010].


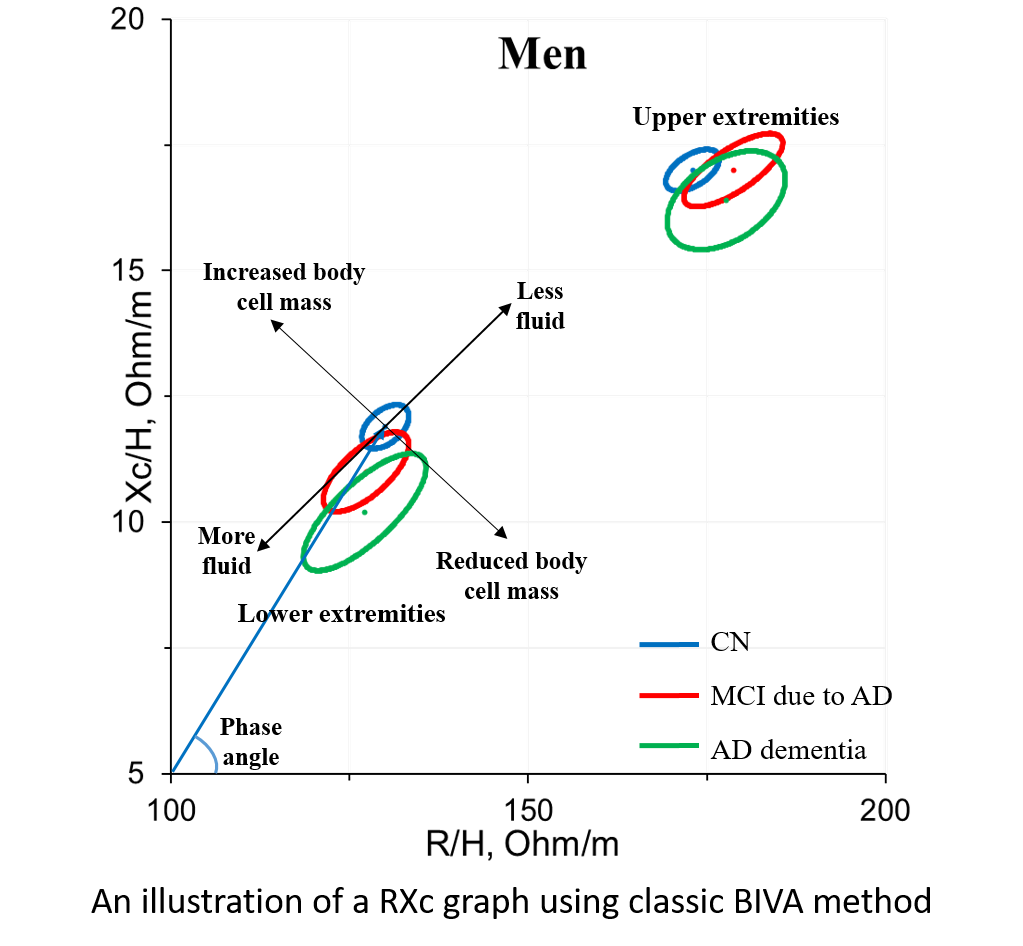


Additional reference:

1. Kasap S.O. Principles of Electrical Engineering Materials and Devices. McGraw-Hill; New York, City, NY, USA: 1997. [[Google Scholar](https://scholar.google.com/scholar_lookup?title=Principles+of+Electrical+Engineering+Materials+and+Devices&author=S.O.+Kasap&publication_year=1997&)]
2. Khalil, S. F., Mohktar, M. S., & Ibrahim, F. (2014). The theory and fundamentals of bioimpedance analysis in clinical status monitoring and diagnosis of diseases. Sensors (Switzerland), 14(6), 10895–10928. https://doi.org/10.3390/s140610895
3. Kyle UG, Bosaeus I, De Lorenzo AD, et al. Bioelectrical impedance analysis--part I: review of principles and methods. Clin Nutr. 2004; 23(5):1226-1243. doi:10.1016/j.clnu.2004.06.004
4. Tanaka S, Ando K, Kobayashi K, et al. Low Bioelectrical Impedance Phase Angle Is a Significant Risk Factor for Frailty. Biomed Res Int. 2019; 2019:6283153. Published 2019 Jun 10. doi:10.1155/2019/6283153
5. Kubo Y, Noritake K, Nakashima D, Fujii K, Yamada K. Relationship between nutritional status and phase angle as a noninvasive method to predict malnutrition by sex in older inpatients. Nagoya J Med Sci. 2021;83(1):31-40. doi:10.18999/nagjms.83.1.31
6. Hirose S, Nakajima T, Nozawa N, et al. Phase Angle as an Indicator of Sarcopenia, Malnutrition, and Cachexia in Inpatients with Cardiovascular Diseases. J Clin Med. 2020;9(8):2554. Published 2020 Aug 6. doi:10.3390/jcm9082554
7. Raimann JG, Abbas SR, Liu L, et al. Agreement of single- and multi-frequency bioimpedance measurements in hemodialysis patients: an ancillary study of the Frequent Hemodialysis Network Daily Trial. Nephron Clin Pract. 2014; 128(1-2):115-126. doi:10.1159/000366447
8. InBody S10. InBody370 USER’S MANUAL. (1996). Available online at:<https://nl.inbody.com/wp-content/uploads/2019/01/InBodyS10_CDmanual_Eng_E.pdf>

(accessed March 15, 2020). [[Google Scholar](https://scholar.google.com/scholar_lookup?journal=InBody370+USER%E2%80%99S+MANUAL.&publication_year=1996&)]

1. Olde Rikkert, M. G. M., Deurenberg, P., Jansen, R. W. M. M., Van’t Hof, M. A., & Hoefnagels, W. H. L. (1997). Validation of multi-frequency bioelectrical impedance analysis in detecting changes in fluid balance of geriatric patients. In Journal of the American Geriatrics Society (Vol. 45, Issue 11, pp. 1345–1351). <https://doi.org/10.1111/j.1532-5415.1997.tb02934.x>
2. Piccoli A, Rossi B, Pillon L, Bucciante G. A new method for monitoring body fluid variation by bioimpedance analysis: the RXc graph. Kidney Int. 1994;46(2):534–9. <https://doi.org/10.1038/ki.1994.305>.
3. Rossini-Venturini, A.C., Abdalla, P.P., Fassini, P.G. *et al.* Association between classic and specific bioimpedance vector analysis and sarcopenia in older adults: a cross-sectional study. *BMC Sports Sci Med Rehabil* **14**, 170 (2022). <https://doi.org/10.1186/s13102-022-00559-2>
4. Buffa, R., Mereu, R. M., Putzu, P. F., Floris, G., & Marini, E. (2010). Bioelectrical impedance vector analysis detects low body cell mass and dehydration in patients with Alzheimer’s disease. Journal of Nutrition, Health and Aging, 14(10), 823–827. <https://doi.org/10.1007/s12603-010-0115-9>
